# A prognostic model for melanoma patients on the basis of immune-related lncRNAs

**DOI:** 10.18632/aging.202730

**Published:** 2021-03-06

**Authors:** Yao Wang, Hong-Jun Ba, Xi-Zhi Wen, Min Zhou, Can Küçük, Luca Tamagnone, Li Wei, Hua You

**Affiliations:** 1 Medical Oncology Department, Affiliated Cancer Hospital and Institute of Guangzhou Medical University, Guangzhou 510095, Guangdong, China; 2 Pediatric Cardiology Department, Heart Center, The First Affiliated Hospital of Sun Yat-Sen University, Guangzhou 510080, Guangdong, China; 3 Biotherapy Center, Sun Yat-Sen University Cancer Center, State Key Laboratory of Oncology in South China, Guangzhou 510060, Guangdong, China; 4 İzmir Biomedicine and Genome Center (IBG), İzmir 35340, Turkey; 5 İzmir International Biomedicine and Genome Institute (iBG-İzmir), Dokuz Eylül University, İzmir 35340, Turkey; 6 Department of Medical Biology, Faculty of Medicine, Dokuz Eylül University, İzmir 35340, Turkey; 7 Department Life Sciences and Public Health, Università Cattolica del Sacro Cuore, Rome 00168, Italy; 8 Fondazione Policlinico Universitario Agostino Gemelli, IRCCS, Rome 00168, Italy

**Keywords:** melanoma, lncRNA, prediction model, gene expression profile, immunotherapy

## Abstract

The prognosis of melanoma patients is highly variable due to multiple factors conditioning immune response and driving metastatic progression. In this study, we have correlated the expression of immune-related lncRNAs with patient survival, developed a prognostic model, and investigated the characteristics of immune response in the diverse groups. The gene expression profiles and prognostic information of 470 melanoma patients were downloaded from TCGA database. Significantly predictive lncRNAs were identified by multivariate Cox regression analyses, and a prognostic model based on these variables was constructed to predict survival. Kaplan-Meier curves were plotted to estimate overall survival. The predictive accuracy of the model was evaluated by the area under the ROC curve (AUC). Principal component analysis was used to observe the distribution of immune-related genes. CIBERSORT and ESTIMATE were used to evaluate the composition of immune cells and the immune microenvironment. Eight immune-related lncRNAs were determined to be prognostic by multivariate COX regression analysis. The patient scores were calculated and divided into high- and low-risk groups. The model could effectively predict the prognosis in patients of different stages. The AUC of the model is 0.784, which was significantly higher than that of the other variables. There were significant differences in the distribution of immune-related genes between two groups; the immune score and immune function enrichment score were higher in the low risk group.

## INTRODUCTION

Melanoma is a highly malignant tumor originating from melanocytes; its prevalence lately shows a global annual increase of about 3%-7% [1]. The prognosis is favorable when the disease is detected at an early stage (stages I and II); however, the prognosis of advanced diseases (stages III and IV) is extremely poor, and the 5-year survival rate on conventional treatment (including chemotherapy and biotherapy) is only 5-10% [2]. Melanoma development is strongly influenced by inflammation and immune cell infiltration [3]. In fact, immune checkpoint inhibitors (ICIs), especially anti-PD-1 and anti-PD-L1 blockers, have changed the traditional treatment mode and achieved unprecedented long-lasting responses in patients with melanoma. However, the effective rate of anti-PD-1 blockade fluctuates between 20% and 40%, and the complete response rate is only about 5% [4]. Therefore, risk stratification of melanoma patients by the combination of immune-related factors and pathological classification is key to predict the prognosis and treatment response.

Long noncoding RNAs (lncRNAs) refer to RNA molecules longer than 200 nucleotides which lack protein coding function. lncRNAs play an extensive regulatory role in different stages of tumor immune response, such as antigen exposure, antigen recognition, immune activation, immune cell infiltration, and tumor clearance [5]. Previous studies showed that a variety of lncRNAs, such as UCA1, DSCAM-AS1 and MIR155HG, can promote melanoma development and are potential therapeutic targets [6–8]. However, the role of immune-related lncRNAs in the prognosis of melanoma is still unclear. In this study, gene expression profiles from the TCGA database were analyzed to establish a relationship between immune-related lncRNAs and the prognosis of melanoma patients followed by construction of a new prognostic model.

## RESULTS

### Characteristics of patients

**Figure 1:**
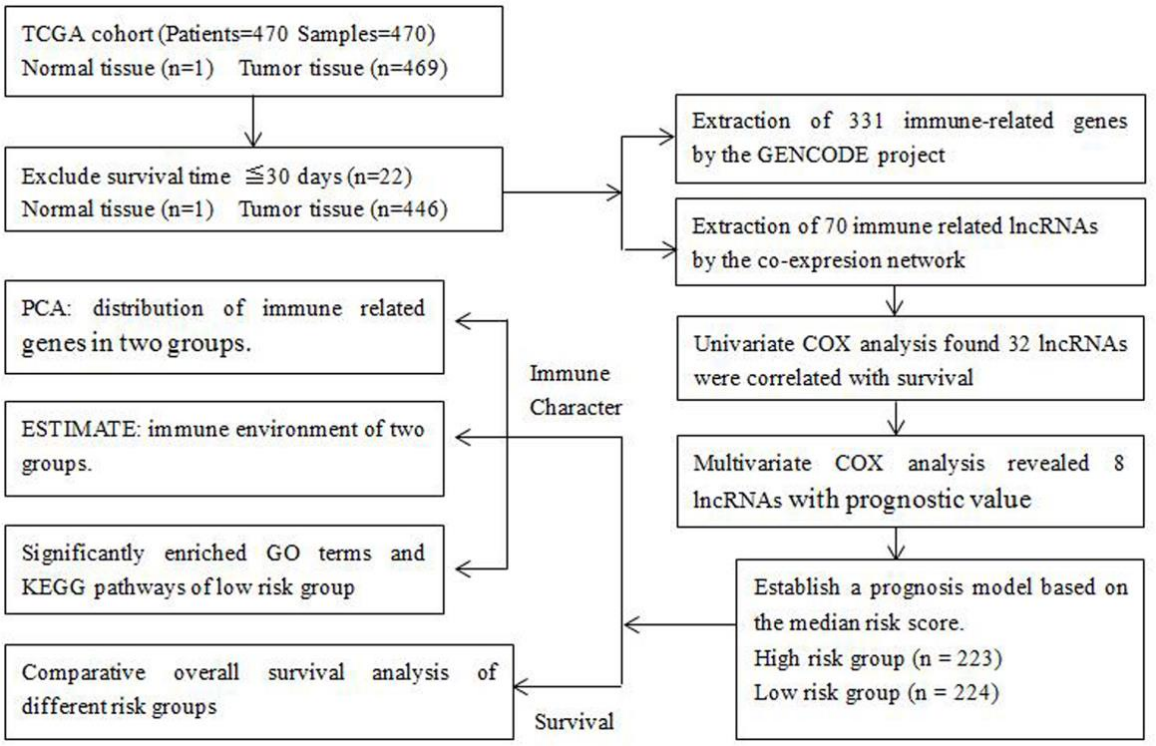
**Flowchart of the detailed study design and samples at each stage of the analysis.**

A total of 470 melanoma patients were analyzed in this study, including 290 males (61.7%) and 180 females (38.3%), with an average age of 59.2 years (18-90 years). The clinicopathological features taken into consideration consisted of age, gender, T stage, lymph node involvement, distant metastasis and clinical stage (Table 1). We drew a flowchart to illustrate the study design and the type of samples involved and analyzed at each step, where each sample represents one melanoma patient (Figure 1).

**Table 1:** Baseline characteristics of patients with melanoma.

**Characteristics**	**N(%)**
Age	
<60	241(51.3)
≥60	221(47.0)
unknown	8(1.7)
Gender	
Male	290(61.7)
Female	180(38.3)
AJCC stage	
I	77(16.4)
II	140(29.8)
III	171 (36.4)
IV	23(4.9)
unknown	59(12.5)
Tumor	
T0	23(4.9)
T1	42(8.9)
T2	78(16.6)
T3	90(19.1)
T4	153(32.6)
unknown	84(17.9)
Lymph Node	
N0	235(50.0)
N1	74(15.7)
N2	49(10.4)
N3	55(11.7)
Nx	57(12.2)
Metastasis	
M0	418(88.9)
M1	24(5.2)
Mx	28(5.9)

### Prognostic significance of the immune-related lncRNAs in melanoma

Three hundred and thirty-one immune-related genes were extracted from the TCGA data set based on the GSEA immune-related gene sets (Immune system process M13664, Immune response M19817). Sixty immune-related lncRNAs were identified with the co-expression method (correlation coefficient Cor>0.7, P<0.001). Furthermore, thirty two immune-related lncRNAs previously associated with the prognosis of melanoma were screened by univariate COX regression analysis (Table 2).

**Table 2:** Univariate COX analysis of the immune-related lncRNAs associated with the prognosis of melanoma.

**LncRNA**	**HR**	**P**
LINC02446	0.67	<0.001
PCED1B-AS1	0.71	<0.001
AC008105.3	0.58	<0.001
MIAT	0.54	<0.001
AC004847.1	0.62	0.001
TRG-AS1	0.62	<0.001
AC136475.3	0.66	<0.001
AC243960.1	0.63	<0.001
USP30-AS1	0.59	<0.001
U62317.1	0.54	<0.001
AL359076.1	0.65	<0.001
LINC02560	1.38	<0.001
TRBV11-2	0.66	<0.001
AC090559.1	0.63	<0.001
C5orf56	0.36	<0.001
DBH-AS1	0.62	<0.001
MIR205HG	1.35	<0.001
AL133371.2	0.56	<0.001
LINC01871	0.72	<0.001
AC004687.1	0.70	<0.001
HLA-DQB1-AS1	0.63	<0.001
PSMB8-AS1	0.74	<0.001
AC018755.4	0.63	<0.001
LINC01943	0.50	<0.001
AC022706.1	0.58	0.001
AC098613.1	0.51	<0.001
ITGB2-AS1	0.65	<0.001
AC015911.3	0.47	<0.001
AC011899.2	0.46	<0.001
AC012236.1	0.62	<0.001
AL590764.1	0.51	<0.001
AL365361.1	0.61	<0.001

### Establishment of a prognostic model

**Figure 2:**
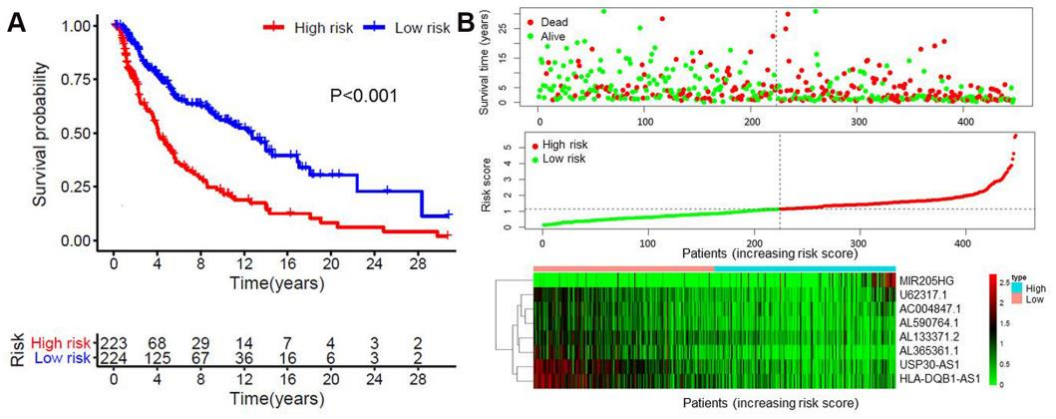
**Construction of the eight immune-related lncRNA risk model of melanoma.** (**A**) Kaplan-Meier survival curve of the prognostic model; (**B**) Risk score distribution, survival status and expression of eight immune-related lncRNAs in high-risk and low-risk groups. Red: high expression; Green: low expression.

Multivariate analysis was performed on the aforementioned 32 immune-related lncRNAs. Eight of 32 lncRNAs were finally identified based on the AIC value, and they were used to construct the prognostic model (Table 3). The risk score of each patient was calculated according to the expression level of each lncRNA and its corresponding regression coefficient. Patients were divided into a high-risk (n = 223) and a low-risk group (n = 224), based on the median risk score. The survival rate was significantly different between two groups (P < 0.001, Figure 2A). The 5-year survival rate of patients in the high- and low-risk groups was 42.7% and 73% respectively. The risk score of each patient and the expression levels of eight immune-related lncRNAs are shown in Figure 2B.

**Table 3:** Immune-related lncRNAs identified through Cox regression analysis.

**LncRNA**	**β**	**HR**	**P. value**
AC004847.1	0.63	1.88	0.022
USP30-AS1	-0.52	0.60	0.007
U62317.1	-0.58	0.56	0.035
MIR205HG	0.43	1.54	<0.001
AL133371.2	-0.33	0.72	0.019
HLA-DQB1-AS1	-0.25	0.78	0.048
AL590764.1	0.92	2.51	0.038
AL365361.1	-0.32	0.72	0.040

### Verification and validation of the prognostic model

**Figure 3:**
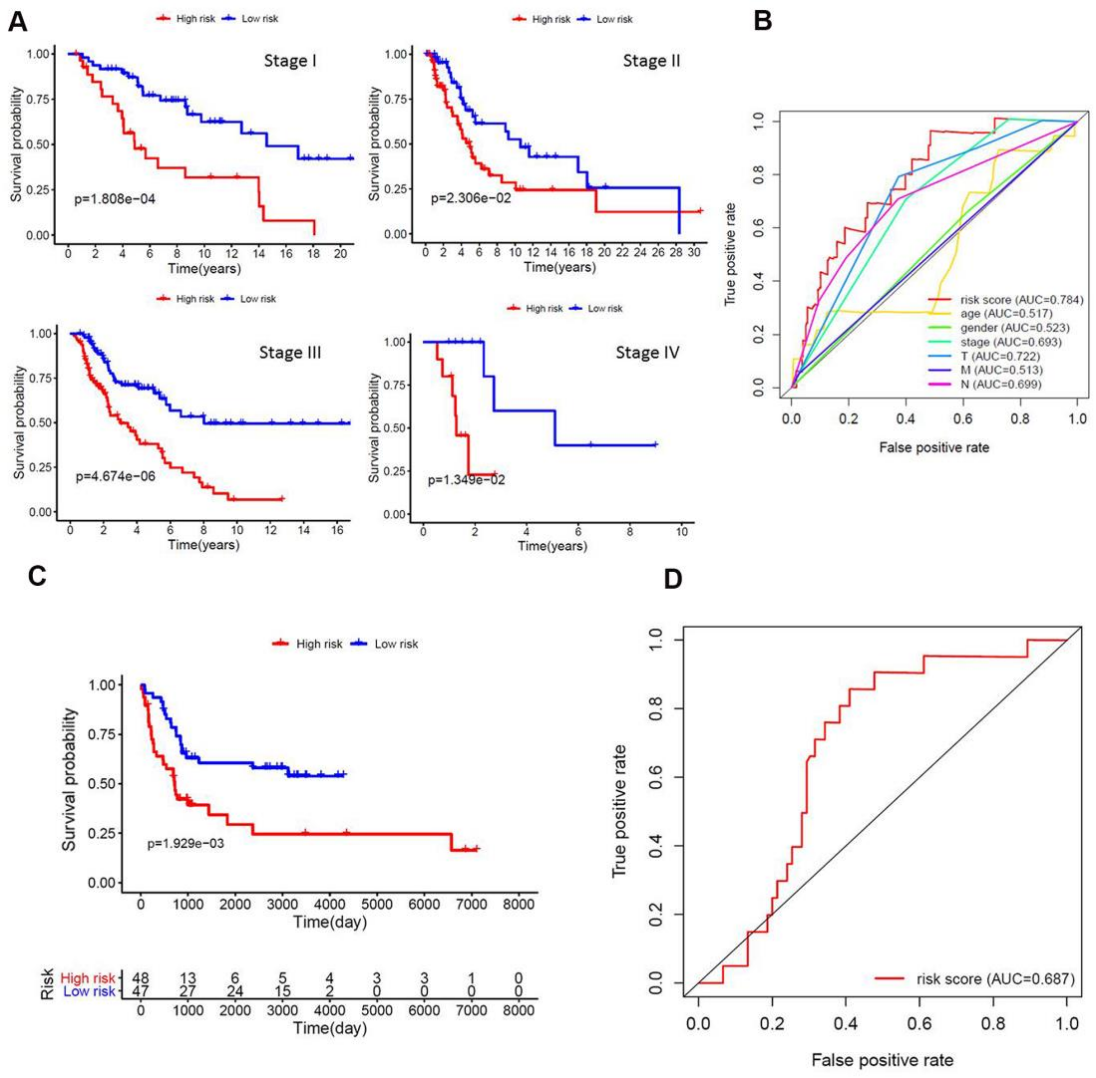
**Functional validation of the prognostic model.** (**A**) Kaplan–Meier analysis according to the prognostic model in melanoma patients of different tumor stage; (**B**) Comparison of ROC curves for prediction of survival by the risk score and other variables (age, gender, stage, tumor, lymph node, metastasis). (**C**) The gene expression profiles of 95 melanoma patients were mined to extract the expression levels of eight lncRNAs and validate the prognostic model using the same algorithm. We also observed significant differences in survival between the two risk groups; (**D**) The area under the ROC curve (AUC) of the validation dataset was 0.687.

To further evaluate the ability of the prognostic model in stratification of patients with different TNM stages, we performed survival analyses and found that the model could effectively differentiate the prognosis among patients of stage I (*P*<0.001), stage II (*P*=0.023), stage III (*P*<0.001), and stage IV (*P*=0.013) (Figure 3A). The area under the ROC curve (AUC) of the prediction model was 0.784 (Figure 3B). In addition, the gene expression profiles of 95 melanoma patients derived from two public datasets (44 samples from GSE98394 and 51 samples from GSE91061) were mined to extract the expression levels of eight lncRNAs and validate the prognostic model using the same algorithm. Of significance, we also observed significant differences in survival between the two risk groups (Figure 3C). The area under the ROC curve (AUC) of the validation dataset was 0.687 (Figure 3D). Our validation analysis demonstrated that this prognostic model can be extended and applied to multiple panels of melanoma cancer patients.

### Immune characteristics of high- and low-risk groups

**Figure 4:**
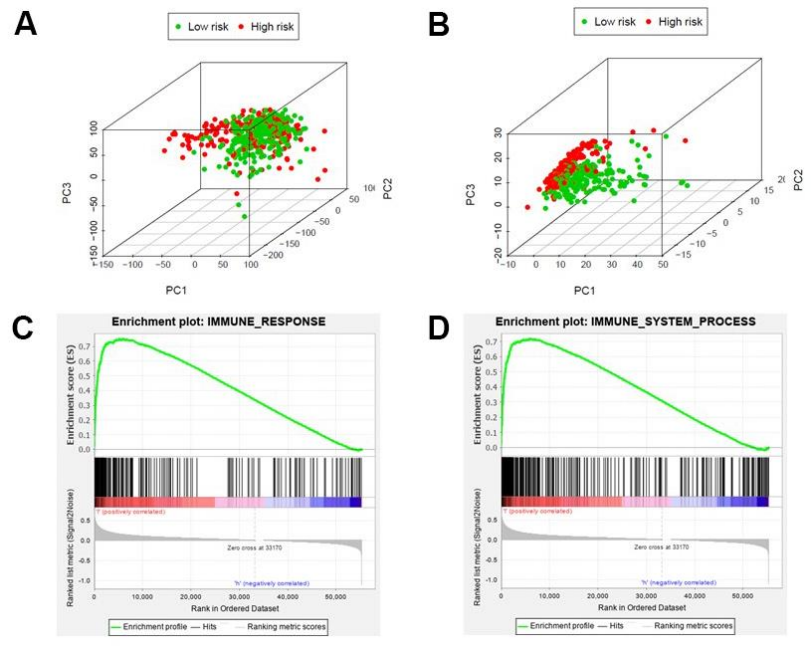
**Different immune status in high-risk and low-risk groups.** Principal components analysis between high-risk and low-risk groups based on all genes (**A**) or immune-related genes (**B**). Enrichment analysis of genes related to immune response (**C**) or immune system process (**D**), which shows gene sets enriched in the low-risk group. NSE: Normalized Enrichment Score.

**Figure 5:**
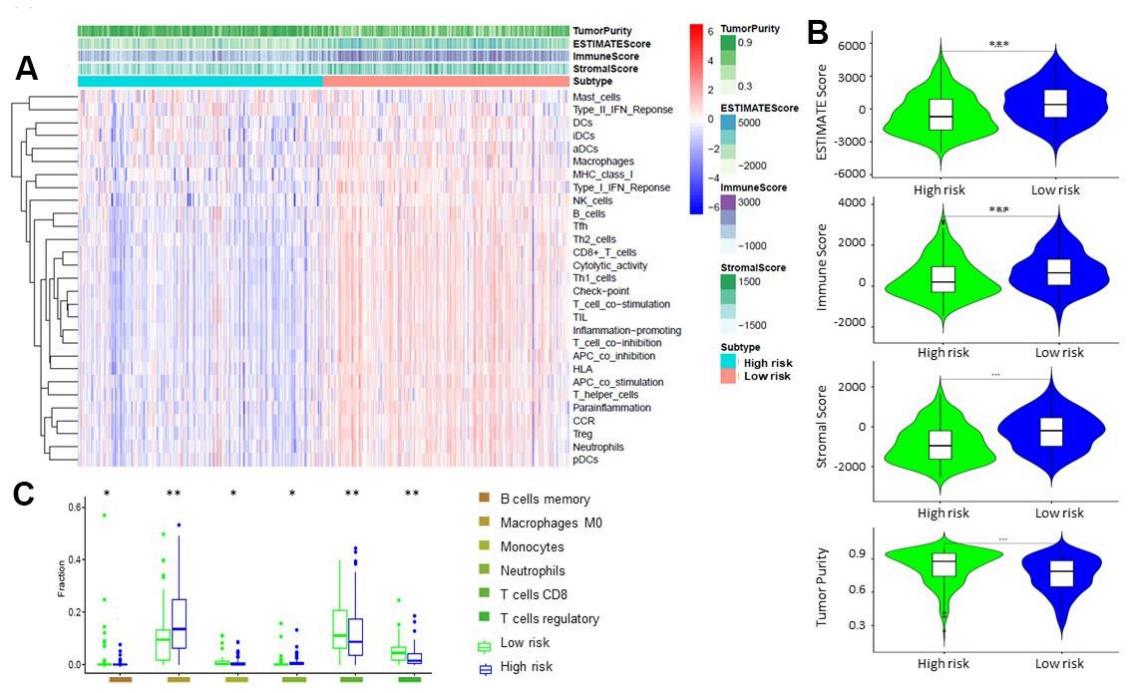
**Comparison of ESTIMATE between high-risk and low-risk groups.** (**A**, **B**) Tumor purity, stromal score, and immune score of different groups were evaluated by ESTIMATE. (**C**). Comparison of TIICs levels between two groups (ANOVA test).

By principal component analysis (PCA) we found that there were significant differences in the expression profile of immune-related genes within the high- and low-risk groups (Figure 4A, 4B). Functional annotation was further performed by GSEA, revealing that differentially expressed genes between the two groups were enriched in immune system process and immune response pathway signatures (Figure 4C, 4D). After performing the ESTIMATE analysis, we observed that the immune score of the low-risk group was higher than that of the high-risk group (Figure 5A). The low-risk group contained a higher proportion of immune cells and stromal cells; however, the tumor purity is relatively low (P<0.001) (Figure 5B). In the low-risk group, CD8^+^ T cells were relatively abundant, whereas a lower proportion of M0 macrophages were detected (Figure 5C). Moreover, GO and KEGG enrichment analysis revealed that the biological functions associated the low risk group were mainly concentrated in immune related functions (Table 4).

**Table 4:** Significantly enriched GO terms and KEGG pathways in the low-risk group.

**GO**	**ID**	**Description**	**NSE**	**p.adjust**
BP	GO:0045088	Regulation of innate immune response	2.65	<0.001
BP	GO:0031349	Positive regulation of defense response	2.63	<0.001
BP	GO:0002218	Activation of innate immune response	2.61	<0.001
BP	GO:0032088	Negative regulation of NF-κB transcription factor activity	2.57	<0.001
BP	GO:0009615	Response to virus	2.57	<0.001
KEGG	hsa05416	Viral myocarditis	2.56	<0.001
KEGG	hsa04630	JAK-STAT signaling pathway	2.46	<0.001
KEGG	hsa04650	Natural killer cell-mediated cytotoxicity	2.46	<0.001
KEGG	hsa04062	Chemokine signaling pathway	2.43	<0.001
KEGG	hsa04612	Antigen processing and presentation	2.43	<0.001

BP, biological process; CC, cellular component; GO, gene ontology; MF, molecular function.

## DISCUSSION

The immune system is involved in recognizing and eliminating tumor cells. They can evade this surveillance through immune escape and immunosuppression; indeed, an abnormal immune response is closely associated with tumor development [9]. In recent years, studies have found that lncRNAs play an important regulatory role in the immune response, especially through the following mechanisms: i) they regulate hematopoietic stem cells’ differentiation in the bone marrow into specific mature immune cell populations [10]; ii) they are involved in the peripheral differentiation of dendritic cells, NK cells and innate lymphocytes [11]; iii) they modulate the immune response by controlling the expression of immune-related genes, toll-like receptors (TLRs), and cytokine receptors [12]. iv) they regulate differentiation and activation of T cells and B cells [13]; v) they participate in the activation of autophagy, and other inflammation-associated processes [14]. Immune-related lncRNAs have predictive value for survival and prognosis of a variety of tumors and are potential targets for cancer treatment [15, 16].

In this study, we performed a genome-wide analysis of expression data derived from 470 melanoma patients found in TCGA database, and explored the relationship between immune-related lncRNAs and prognosis of melanoma through survival analysis and Cox regression model. Among the eight immune-related lncRNAs selected to construct the prognostic model, MIR205HG, AC004847.1 and AL590764.1 were poor outcome risk genes, while U62317.1, USP30-AS1, AL133371.2, AL365361.1 and HLA-DQB1-AS1 were protective genes. Previous studies have identified MIR205HG as a potential therapeutic target, as it can promote tumor development and progression by regulating gene transcription [17]; while other lncRNAs have not been characterized previously by relevant clinical or basic studies, and their mechanism of action is still unclear, warranting future investigation.

The incidence and clinical features of melanoma, such as pathological classification, anatomical site and prognosis, are significantly different in different ethnic groups [18]; and the prognosis of patients defined at the same stage is also different. Therefore, a better understanding of the prognostic factors of melanoma is needed. In the proposed model, patients were divided into high- and low-risk groups, and the survival rate between the two groups showed statistically significant differences. AUC (area under the ROC curve) of this prognostic model was 0.784, which was significantly higher than those of the other clinicopathological factors, such as age, gender, T stage, lymph node involvement, distant metastasis and clinical stage. Furthermore, this prognostic model can effectively stratify the risk of melanoma patients belonging to different stages, which indicate that it can be used as a useful complement to the TNM staging system.

Immune checkpoint inhibitors are used as a major therapeutic option for melanoma patients in advanced stage; however, their curative effect depends on the immunogenicity of the tumor. At present, the predictive markers of ICIs in melanoma treatment include the expression level of PD-L1 in tumor cells, the tumor mutation burden (TMB), the presence of tumor infiltrating lymphocytes (TIL), the presence of insertion and deletion (indel) mutations, the number of POLE mutations, specific gut microbiota, etc. [19]. Of course, the characteristics of the tumor microenvironment and of infiltrating lymphocytes play a major role in the process of anti-tumor immunity [20]. In this study, we found that the tumor microenvironment of melanomas in the low-risk group contained larger number of immune cells and stromal cells and lower number of tumor cells. Studies have shown that CD8^+^T cells, as important effector T cells for tumor elimination, can help to prolong the overall survival of patients and improve the efficacy of immunotherapy [21]. Tumor-associated macrophages (TAM) can express enzymes and cytokines that may be involved in inhibition of the recruitment and activation of T cells, thereby leading to resistance to ICI drugs. Recently, many ongoing clinical trials focus on therapies that inhibit the proliferation or polarization of TAMs to further enhance the anti-tumor immune response [22]. Consistent with previous studies, we found that the low-risk group with higher proportion of CD8^+^ T cells and lower proportion of M0 macrophages had better prognosis. GO and KEGG enrichment analyses showed that immune-related functions were represented mostly in this group. There were significant differences in terms of expression levels of the immune-related genes in two groups. Of note, the immune score and the PD-L1 expression levels were higher in the low-risk group. Based on the GSEA, the low-risk group contained pathways related to immune response and immune system process. Thus, the low-risk group might be more suitable for immunotherapy as a result of high immunogenicity. Nevertheless, the mechanism is still unclear. Eventually, the response rate to ICIs in different risk groups need to be further evaluated in future studies to validate our findings.

In summary, we analyzed the relationship between immune-related lncRNAs and survival status of melanoma patients and constructed a model to predict the prognosis of patients. There were significant differences in immune status between the high-risk and the low-risk patient group, which may also be applied as a biomarker to evaluate their suitability for immunotherapy. The establishment of this model may thus become convenient for clinicians to choose the most appropriate treatment. We validated this model using two public datasets and their corresponding survival data. Although we observed significant statistical difference of survival outcomes between the high- and low-risk groups, the relatively low frequency of melanoma cancer patients limits the opportunities of validation using additional cohorts. The application value of this proof-of-concept study could be further validated by multi-centric clinical studies including high number of patients.

## MATERIALS AND METHODS

### Data collection

The gene expression profile and corresponding prognosis information of 470 patients with melanoma derived tumor and normal tissues were downloaded from the TCGA database (The Cancer Genome Atlas, https://cancergenome.nih.gov/). Patients with the survival time less than 30 days were excluded from further analyses because they may have died of other fatal complications. Ultimately, 447 patients were enrolled in our research and each tumor case corresponded to one independent patient. Data collection time: October 1, 2019.

### Extraction of immune-related lncRNAs

Methods for the extraction of immune related lncRNAs have been described in previous studies [23, 24]. The immune-related gene sets (Immune system process M13664, Immune response M19817) were downloaded from GSEA web site (http://software.broadinstitute.org/gsea/index.jsp) [25]. We obtained the expression levels of immune genes and lncRNAs in each sample, followed by identification of a co-expression cohort of immune-related lncRNAs through Pearson’s correlation analysis by the cor.test function of R (correlation coefficient Cor>0.8, P<0.001).

### Prognostic model construction and validation

The significance of lncRNAs for survival was analyzed using the Cox proportional hazards model by the Survival package of R (3.5.2) software. Univariate Cox analysis showed that expression of 32 lncRNAs were correlated with survival. Multivariate Cox analysis of these 32 lncRNAs revealed 8 lncRNA with significant prognostic value. We fitted all models with different variable combinations, and used Akaike Information Criterion (AIC) to evaluate goodness of fit (GOF) of each model. Finally, we selected AIC to build the model with the smallest variable. The risk score of each patient was determined according to the lncRNA expression level and the regression coefficient (β) of the weighted linear combination in the multivariate analysis. The calculation formula of risk score for each patient is as follows: Risk score =βgene1×expr(gene1)+ βgene2×expr(gene2)+...+ βgeneN×expr(geneN), (expr: lncRNA expression level), β: regression coefficient). The median risk score was used to divide the group into a low-risk and a high-risk group. To evaluate the accuracy of the prognostic model, the same algorithm was applied in the validation sets (GSE91061 and GSE98394) with survival outcomes. Melanoma patients’ mRNA sequencing data from GSE91061 and GSE98394 were downloaded using the “prefetch” software in SRA format. The SRA files were converted to fastq format data by “fastq-dump” program. “fastqc” was used to control quality. “trim_galore” was used to cut adapt. “hisat2” and “samtools” were used to generate bam files. “featureCounts” was used to get the counts of each genes. After including the clinical survival data, 44 samples from GSE98394 and 51 samples from GSE91061 were screened out for next analysis. “SVA” package in R program was used to move the batch effect in combining the two datasets.

### Tumor component assessment

We use the CIBERSORT analytical tool to identify the relative percentages of 22 types of tumor infiltrating immune cells (TIICs) with normalized gene expression data [26]. The gene sets of twenty nine immune markers were defined according to the function of the immune genome [27]. The ESTIMATE (Estimation of STromal and Immune cells in MAlignant Tumors using Expression data) program was used to evaluate the immune score, stromal cell content, and tumor purity of each sample [28].

### Statistical analysis

Overall survival (OS) was defined as follows: the time passed from the date of diagnosis to the end of the follow-up period, to the date of death from any cause, or to the date when patient cannot be followed-up anymore. Kaplan–Meier curves were plotted to estimate overall survival; and the log rank test was performed to evaluate statistical significance of differences in survival. Univariate and multivariate analysis was performed by Cox proportional hazards model. ROC curves and area under the curve (AUC) were calculated to determine their predictive value. Principal component analysis (PCA) was apply to observe the clustering of immune-related genes belonging to the two groups. All statistical analyses were carried out using R (3.5.2) software and P<0.05 (bilateral) was defined as a statistical difference.
